# Empirical Research on the Intervention of Ankylosing Spondylitis With Tai Chi

**DOI:** 10.1093/geroni/igaf122.1830

**Published:** 2025-12-31

**Authors:** Sinuo Chen

**Affiliations:** The School of the Art Institute Of Chicago, China (People’s Republic)

## Abstract

This study aims to explore the intervention effect of Tai Chi on patients with ankylosing spondylitis. The experimental group received a 12-week Tai Chi intervention. The Tai Chi exercise prescription was designed based on the pathological characteristics of ankylosing spondylitis. The study indicates that the Tai Chi exercise prescription intervention can effectively improve the symptoms and functions of patients with ankylosing spondylitis and enhance their quality of life.

